# Ten years left to eliminate blinding trachoma

**Published:** 2010-09

**Authors:** Danny Haddad

**Affiliations:** Director, International Trachoma Initiative, 325 Swanton Way, Decatur, GA 30030 USA.

In 1997, the World Health Organization formed the Global Alliance to Eliminate Blinding Trachoma by 2020 (GET 2020), a coalition of governmental, non-governmental, research, and pharmaceutical partners. In 1998, the World Health Assembly urged member states to map blinding trachoma in endemic areas, implement the SAFE strategy (which stands for surgery for trichiasis, antibiotics, facial-cleanliness and environmental change, such as clean water and latrines) and collaborate with the global alliance in its work to eliminate blinding trachoma.

Over these past 13 years, much progress has been made. Pfizer Inc has committed to donating the Zithromax necessary for eliminating blinding trachoma by 2020, non-governmental organisations have scaled up their support to national programmes to implement the SAFE strategy, and some trachoma-endemic countries are now close to reaching their intervention goals. Since the Pfizer donation began in 1999 through the International Trachoma Initiative (ITI), more than 155 million Zithromax treatments have been distributed.

**Figure 1:**
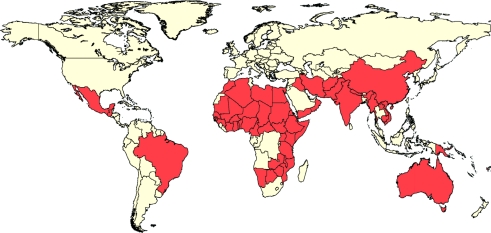
Trachoma-endemic countries

Source: Mariotti SP, Pascolini D, Rose-Nussbaumer J, “Trachoma: global magnitude of a preventable cause of blindness,” British Journal of Ophthalmology 2009:93: 563-568

But trachoma remains a blinding scourge. It is still believed to be endemic in 57 countries (Figure 1). Globally, 1.2 billion people live in trachoma-endemic areas, primarily in the poorest communities in low- and middle-income countries. Nearly 41 million people, mostly women and children, have active trachoma and could benefit from treatment. An estimated 8.2 million already have trichiasis, the end stage of the disease, and are at risk of becoming blind or visually impaired.

We only have ten years left to reach the goal of eliminating blinding trachoma. In order to achieve this, all endemic countries must have the full scale SAFE strategy in place by 2015 to allow enough time for implementation to have an impact. Enormous challenges lie ahead of us. Some of the remaining endemic countries are in conflict or have just come out of conflict, and lack infrastructure and resources to fully address the disease. Even countries free of conflict lack financial resources for the epidemiological surveys to determine which districts need intervention, or to support intervention in endemic districts. Implementing the SAFE strategy can be a strain on resources as well, since providing access to clean water and latrines is not inexpensive.

However, we believe that, together, we can overcome these challenges and reach our goal.

In this issue of the *Community Eye Health Journal,* a series of articles begins on blinding trachoma, which has incapacitated families and communities for centuries in nearly every corner of the world. The goal of this series is to explore what is new in the campaign to eliminate the disease, including recent developments in trichiasis surgery, mass drug administration, and cost-effective ways to improve sanitation and hygiene. Previous editions of the *Community Eye Health Journal* that focused on trachoma (editions 32 and 52) remain important resources for trachoma.

The first article in the series is about national trachoma task forces. We hope that this new series will provide tools to assist those who are implementing programmes to eliminate blinding trachoma.

**Figure:**
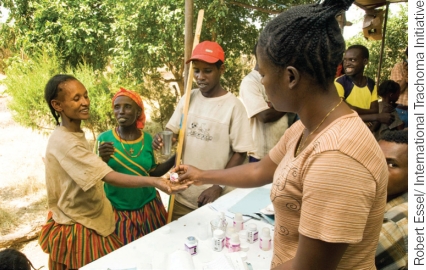
Mass distribution of antibiotics is one of the four components of the SAFE strategy. ETHIOPIA

Robert Essel/International Trachoma Initiative

