# Supplementary material for: Ten years left to eliminate blinding trachoma
Source: Community Eye Health. 2010 Sep;23(73):38. (PMC2975121)
Supplement: Related texts

# Brief report: National trachoma task forces: how can we work better?

**Published:** 2010-09

**Authors:** Paul Courtright, Emmanuel Miri

**Affiliations:** pcourtright@kcco.net; emmamiri@yahoo.com

Tackling trachoma is a complex challenge.

In order to implement all four components of the SAFE strategy on a national level (surgery for trichiasis, antibiotics, facial cleanliness and environmental change, such as clean water and latrines), there needs to be national coordination, supported by political commitment at the highest level. In each trachoma-endemic country, the body responsible for making this work is the national trachoma task force (NTTF).

The NTTF consists of government representatives, NGOs, donors, academic institutions, and other stakeholders. In practical terms, the work of the NTTF involves:

- Devisinga national plan for tackling trachoma that addresses each of the four components of the SAFE strategy and which is based on evidence and technical know-how. Setting national targets and timeline activities for the plan
- Planning and managing different activities within the national plan
- Bringing together the different groups needed to make the national plan work (government, NGOs, donors, etc.); ensuring good communication and trust between the groups and ensuring that everyone has realistic expectations
- Coordinating operational research on trachoma
- Monitoring and evaluating the successes of, and challenges faced by, the national plan, and revising the plan as needed
- Sharing information about the ongoing progress of the national plan with everyone involved, including the public.

Of vital importance to the success of an NTTF is the extent to which it can foster high-level political commitment from the national government.

Simply having a government representative on the NTTF is not enough. These individuals must be ‘doers’ - people who take an active interest and are willing to act and lobby their peers in government on behalf of the NTTF. The reality is that there is always going to be competition within government departments, and between departments, for limited budgets. The NTTF needs to have strong political - and financial - support from within government. The following may help:

- Invite the national director of public health (or a similarly high-level government official) to join the leadership of the NTTF.
- Use visits by experts or other key international leaders as an opportunity to organise meetings with potentially helpful government officials.

Once political commitment to the national plan and the NTTF is achieved, the next goal is for this to be sustained in the long term. This is best achieved by putting systems and processes in place that will ensure that trachoma prevention becomes part of the regular business of government.

Consider doing some or all of the following on a regular basis:

- Develop a trachoma “organogram” showing the various partners (including government) and their activities in the country.
- Provide frequent and professional reports of meetings.
- Disseminate reports, updates, and fact sheets to key government officials on a regular basis.
- Regularly review the current trachoma knowledge base.
- Reach consensus with partners and all districts on how to report on trachoma data or develop a harmonised management information system (MIS) for trachoma in the country.
  Jointly celebrate achievements or special dates - share the credit for tasks accomplished.
- Organise routine meetings with specific government officials; the meetings should include a cross-section of members of the NTTF.
- Share with government feedback from international meetings that one or more NTTF members have attended.

Having a strong, well-functioning, and collaborative NTTF will assist trachoma-endemic countries in sub-Saharan Africa to realise their goal of eliminating blinding trachoma. An NTTF will not achieve this without specific investments in capacity building in the areas of leadership, partnership, management, and political ownership. Make use of every opportunity to build skills in these areas, whether through formal courses or informal sessions during NTTF meetings.

Investments made now will reap benefits beyond the time when trachoma ceases to be a public health problem.
